# A Randomized Control Trial Comparing Common Errors Made by Women During Three Different Methods of Pelvic Floor Muscle Contraction Training: By Verbal Education vs. Vaginal PalpationTraining vs. Perineometer Training

**DOI:** 10.3390/medicina61030477

**Published:** 2025-03-09

**Authors:** Duygu Sultan Öge, Fatma Kılıç Hamzaoğlu, Hanife Doğan, Türkan Akbayrak

**Affiliations:** 1 Fundamental Physiothetapy and Rehabilitation Programme, Graduate School of Health Sciences, Hacettepe University, 06100 Ankara, Turkey; 2 Department of Gynecology and Obstetrics, Meram Medical School, Necmettin Erbakan University, 42080 Konya, Turkey; dr_fatmakilic_78@hotmail.com; 3 Department of Physiotherapy and Rehabilitation, Nezahat Keleşoğlu Faculty of Health Sciences, Necmettin Erbakan University, 42090 Konya, Turkey; hanife_dogan@yahoo.com.tr; 4 Faculty of Physical Therapy and Rehabilitation, Hacettepe University, 06100 Ankara, Turkey; takbayrak@yahoo.com

**Keywords:** biofeedback, electromyography, palpation, pelvic floor, muscle contraction, muscle contraction training

## Abstract

*Background and Objectives*: The aim of this study was to compare the effects of pelvic floor muscle contraction training (PFMCT) using verbal education, digital vaginal palpation (DVP), or perineometer on the common errors made during pelvic floor muscle contraction (PFMC) in women. *Materials and Methods*: A total of 48 women participated, and they were randomly assigned to three groups (Group I: PFMCT with verbal education, *n* = 16; Group II: PFMCT with DVP, *n* = 16; and Group III: PFMCT with perineometer, *n* = 16). Participants who had not previously received PFMCT were evaluated for pelvic floor muscle strength using the Modified Oxford Scale (MOS), and pelvic floor muscle activation was assessed with electromyographic biofeedback (EMG-BF). Possible errors during pelvic floor muscle contraction (gluteal, adductor and/or abdominal muscle contractions, stop breathing (breath holding), enhanced inhaling, and straining) were evaluated through inspection, palpation, or EMG-BF. After pre-training evaluations, all participants received training on pelvic floor. After this general training, each group received PFMCT using the specific training method for their group. After the training, the same evaluations were repeated. The sessions were conducted one-on-one and lasted for an average of one hour. *Results*: After the training, MOS values increased in Group II and Group III, while EMG-BF values only increased in Group II (*p* < 0.05). The number of incorrect movements during PFMC decreased after the training in all three groups (*p* < 0.05). The abdominal muscle contraction value monitored by EMG-BF only decreased in Group II (*p* < 0.05). *Conclusions*: Our study demonstrated that the PFMCT applied using the DVP method was more effective in creating more accurate and stronger muscle contractions and reducing common errors when compared to pre- and post-training values. Significant differences were observed between the groups in terms of performance improvements, with Group II showing the most notable progress. These results support the potential for DVP to yield better outcomes when used in PFMT.

## 1. Introduction

The International Continence Society defines normal pelvic floor muscle contraction as the ability to contract and relax the pelvic floor muscles in a controlled manner [1,2]. Studies indicate that women, even if they have received detailed education about the pelvic floor, have difficulty distinguishing pelvic floor muscle contractions from other muscle contractions. Therefore, it is emphasized that women are often unsure whether they are contracting these muscles correctly [2,3,4]. Studies in the literature highlight that both asymptomatic women and those with pelvic floor dysfunction (PFD) have low success rates in voluntary muscle contractions. It is also mentioned that being able to contract the pelvic floor muscles correctly is as important as the muscle strength itself [4]. Thompson et al. [5] stated that 23% of women perform pelvic floor muscle exercises incorrectly and that pelvic floor muscle contraction should be individually assessed by a healthcare professional experienced in this area. During pelvic floor muscle contraction, some common mistakes can be observed. These mistakes include contracting the abdominal muscles, hip adductor muscles, or gluteal muscles, stop breathing, enhanced inhaling, pelvic tilt, and straining [2,6].

A study in the literature has coined the term COMMOV for “C”ontractions of “O”ther “M”uscles (m. rectus abdominus, the gluteal muscles, and the adductors), and other “MOV”ements (pelvic tilt, breath holding, and straining) [6].

Although pelvic floor muscle training is recommended as a first-line treatment of urinary incontinence and pelvic organ prolapse (level 1A scientific evidence) [7], studies on pelvic floor muscle contraction training techniques are limited. This situation leads to difficulties for physiotherapists in choosing the therapeutic methods they would prefer. In the literature, individuals are provided with muscle contraction training through various assessment methods to help them acquire motor skills [8,9,10].

According to Wulf et al. [11], motor learning is the development of individuals’ physical skills to adapt to environmental changes. This process, which typically occurs through repetition and experience, enables the learned skills to become more efficient, accurate, and automatic. The evolutionary process of motor learning involves the transition from conscious and voluntary control of movements to a more automatic and unconscious control over time. Motor learning can take different forms. When a person focuses on their body movements while performing a motor skill, it is referred to as an internal focus. In contrast, focusing on the effect of the movement through any device or tool is referred to as an external focus. As Wulf et al. [11] mention in their “A Constrained Hypothesis”, when individuals are instructed to focus on an internal aspect, they tend to unintentionally disrupt automatic control processes while trying to control the movement. Studies have shown that when athletes focus on the effect of the movement, their performance improves, particularly in terms of shooting accuracy or overall performance, due to the activation of automatic processes [12,13]. A large-scale meta-analysis has reported that external focus is superior to internal focus [14].

It is observed that studies related to this topic in the literature are generally limited to athletes and students. Accordingly, in our study, we aimed to investigate the effects of different types of focus on motor learning by examining PFMCT with verbal instructions as an internal focus, while also considering PFMCT as an external focus using the DVP and perineometer devices. Thus, we aimed to provide a new perspective on motor learning methods for the pelvic floor in the literature.

To the best of our knowledge, there are no studies in the literature that thoroughly investigate the effects of different teaching methods used in pelvic floor muscle contraction training (PFMCT) on common errors. The aim of this study was to compare the effects of verbal instruction-based PFMCT, DVP-based PFMCT, and perineometer-based PFMCT, and to examine their impact on common errors that occur during pelvic floor muscle contraction.

## 2. Materials and Methods

### 2.1. Design and Participants

This study is a randomized controlled, single-center study. Participants were selected from women attending routine check-ups at a gynecology clinic affiliated with the university. Out of 64 women who visited the clinic, 48 women who volunteered and met the inclusion criteria were included in the study. The study protocol was approved by the Ethics Committee (no: 2023/437) and registered on ClinicalTrials.gov (accessed on 9 February 2025) (ID: NCT06306703). Participants were informed about the study in accordance with the principles outlined in the Helsinki Declaration, and each participant was asked to read and sign an informed consent form. Additionally, this study was financially supported by TÜBİTAK (The Scientific and Technological Research Council of Turkey) 1002A Rapid Support Program (no: 224S541). This study was produced as part of a doctoral thesis.

The inclusion criteria were as follows: being a woman between the ages of 20 and 50, volunteering to participate in the study, scoring 25 or higher on the Mini Mental Test, having the ability to perform pelvic floor muscle contractions (>2 points according to MOS), and not having previously received pelvic floor muscle contraction training. Women who were pregnant, had stage ≥ 3 symptomatic pelvic organ prolapse, had communication problems, could not cooperate, had a urinary tract infection, epilepsy, or associated neurological disorders were excluded from the study.

Each woman participating in the study was individually assessed and received a single training session. The study lasted approximately 60 min. As stated in the inclusion criteria, women who had previously undergone pelvic floor muscle training were not eligible to participate in our study.

### 2.2. Randomization

Participants were equally assigned to each group using a computer-based block randomization procedure prepared by an independent researcher who was not involved in the study.

### 2.3. Assessments

To assess the participants’ cognitive adequacy, the Mini Mental Test [15,16] was first administered, and those who scored 25 or higher were included in the study. Demographic, physical, and obstetric information of the participants was then collected. Following this, participants were assessed prior to pelvic floor muscle contraction training (PFMCT). Participants were asked to empty their bladders prior to the assessments. To ensure complete relaxation of the pelvic floor muscles, reduce the effects of gravity, and eliminate accessory muscles such as the hip adductors, participants were positioned supine with their knees approximately 140° flexed, thighs and feet about 30 cm apart, and the soles of their feet in contact with the bed.

#### 2.3.1. Assessment of Pelvic Floor Muscle Strength with DVP Before PFMCT

Pelvic floor muscle strength with digital vaginal palpation (DVP) was assessed using the Modified Oxford Scale (MOS). It is a method in which the force created by the contraction of the pelvic floor muscles is manually scored by placing the index and middle fingers in the vagina. The scoring is applied on a scale from 0 to 5. At 0, no contraction is felt, while at 5, a strong contraction is felt [17]. The physiotherapist inserted their index and middle fingers into the participant’s vagina approximately 3 to 5 cm (cm). During the procedure, the participant was instructed, “Tighten strongly around the fingers that I have placed in your vagina”. The participants were instructed to squeeze three times, and the highest value obtained was recorded [18,19,20].

#### 2.3.2. Assessment of Pelvic Floor EMG Muscle Activation Before PFMCT

The EMG activity of the participants’ pelvic floor muscles was assessed using an intravaginal EMG probe (Everyway Medical Instruments Co. New Taipei City, 22203, Taiwan) connected to the NeuroTrac^®^ Myo Plus Pro device (Verity Medical, Hampshire, UK). The NeuroTrac Software 5.1.1 was used to link the device to the computer. The EMG reference cable was placed with a surface electrode on the participant’s right thigh. Participants were instructed to “contract and relax the muscles you use to hold urine”. The peak values of maximum voluntary contractions were recorded in microvolts (μV). Measurements were repeated three times, with 5 s of contraction followed by 5 s of relaxation [7,21,22].

#### 2.3.3. Assessment of Errors During PFMCT and Before PFMCT

Six potential errors during pelvic floor muscle contraction (PFMC) were evaluated. These errors were examined using the error and observation methods described by Bø et al. [2]. To specifically assess the co-contraction of abdominal muscles during PFMC, an EMG-BF device was used instead of visual observation. All error recordings were made while participants were performing PFMC.

For tracking the contraction of abdominal muscles, the electrical activity in the obliquus internus abdominis and transversus abdominis muscles was recorded by placing surface electrodes on the medial aspect of the anterior superior iliac spines. During the measurement, the passive electrode was placed on the participant’s right thigh. The EMG NeuroTrac^®^ Myo Plus Pro device (Verity Medical, Hampshire, UK) was used for evaluation [23]. The co-contraction of the abdominal muscles during PFMC was recorded as peak values in microvolts (μV).

Contractions of the hip adductor muscles were monitored by the physiotherapist placing her hand on the adductor muscles. The test was considered positive if a contraction was felt in the inner thigh muscles or if the legs moved closer together. Gluteal muscle contraction was determined by observing the participant’s movement of squeezing and lifting their hips off the bed. Enhanced inhaling was identified when the participant took a deep breath during PFMC, causing the chest to rise excessively during inspiration. Stop breathing was determined by observing the participant closing their mouth and holding their breath. Straining was recorded when the therapist’s fingers or the perineometer probe were pushed during the procedure [2].

### 2.4. Training

**Figure 1:**
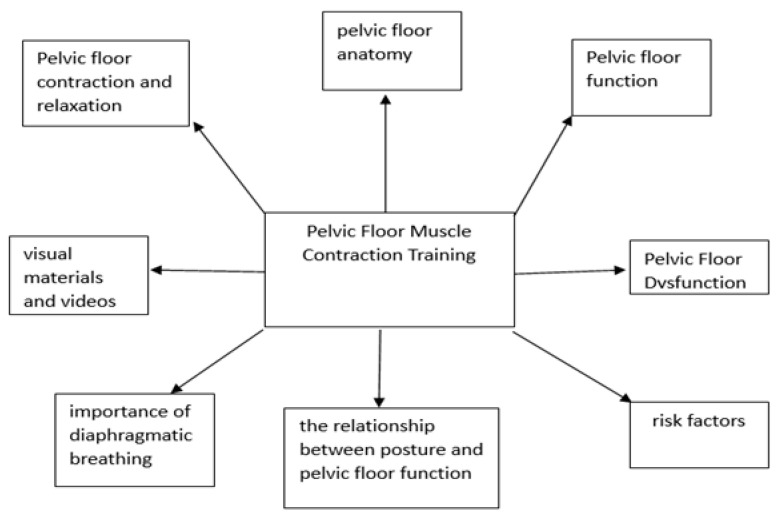
PFMCT.

After the assessments, participants were provided with similar training regardless of the group they were assigned to. The training included detailed presentations on pelvic floor anatomy and function, pelvic floor dysfunction (PFD), risk factors, the relationship between posture and pelvic floor function, importance of diaphragmatic breathing, and pelvic floor muscle training. Additionally, visual materials and videos were used during the training. Pelvic floor contraction and relaxation techniques were explained according to the group to which the participants were assigned (Figure 1).

In Group I, participants received verbal instruction only, without any tactile or visual biofeedback.

In Group II, participants were trained using digital vaginal palpation (DVP), where the physiotherapist placed her index and middle fingers into the participant’s vagina.

In Group III, an intravaginal probe of a perineometer device (Peritron 9300 perineometer-Laborie, Mississauga, ON, Canada) was placed in the participants’ vaginas. The display section of the device was handed to the participant [24]. This allowed for visual biofeedback through numerical values generated by the contraction and relaxation of the pelvic floor muscles.

### 2.5. Post-PFMCT Assessments

After the pelvic floor muscle contraction training (PFMCT), the assessments were repeated. These included the evaluation of muscle strength using digital vaginal palpation (DVP) (MOS), the assessment of pelvic floor muscle activation (EMG-BF), and the evaluation of errors during pelvic floor muscle contraction (PFMC).

### 2.6. Sample Size Estimation

The sample size was calculated using the G-Power 3.1.9.4 program, taking into account the significance level of the hypothesis and the effect size. The effect size of 0.47, obtained from the reference study [25], was used. To detect a significant difference in the study, with α = 0.05 and 1 − β = 0.80, the sample size for each group was calculated to be a minimum of 16 participants (total of 48 women) with 80% power.

### 2.7. Statistical Analysis

The data were analyzed using SPSS 25.0 (IBM SPSS Statistics 25 software, Armonk, NY, USA: IBM Corp.). Continuous variables were presented as mean ± standard deviation, median (range), and categorical variables as frequency and percentage. The normality of the data was assessed using the Shapiro–Wilk test. When the assumptions of parametric tests were met, independent group differences were compared using one-way analysis of variance (post hoc: Tukey test). When the assumptions of parametric tests were not met, independent group differences were compared using the Kruskal–Wallis variance analysis (post hoc: Bonferroni-corrected Mann–Whitney U-test). Differences between categorical variables were examined using the chi-square test. For pre- and post-training comparisons of categorical variables, the McNemar test was used. For continuous variables, when parametric test assumptions were met, dependent group differences were assessed using the paired *t*-test, and when parametric assumptions were not met, the Wilcoxon signed-rank test was used. A *p*-value of <0.05 was considered statistically significant in all analyses.

## 3. Results

**Figure 2:**
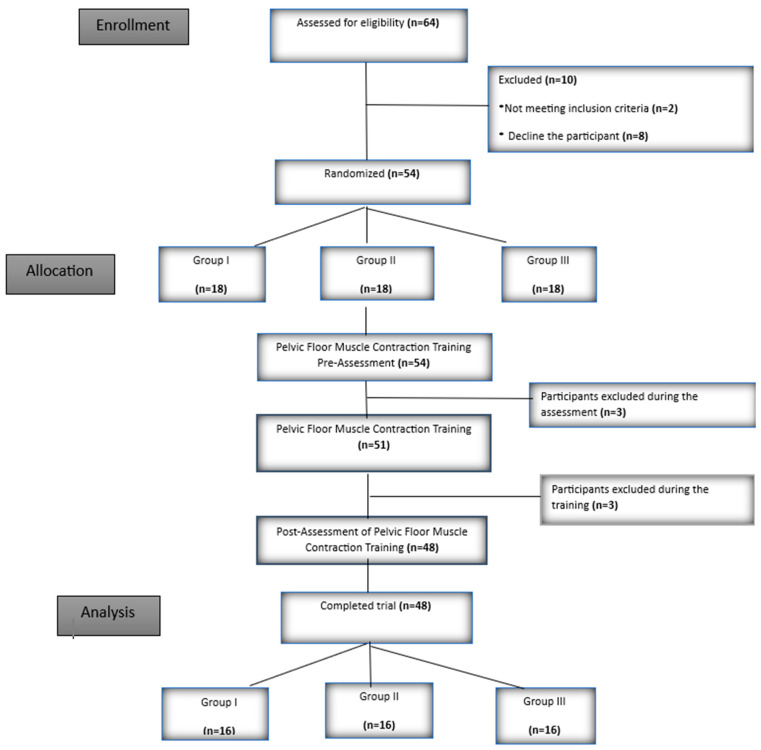
Flow chart.

The study took place between 1 April 2024 and 15 June 2024. A total of 48 participants completed the trial, divided into Group I (*n* = 16), Group II (*n* = 16), and Group III (*n* = 16) (Figure 2).

### 3.1. Baseline Characteristics

The demographic, physical, and obstetric characteristics, educational status, and type of delivery of the participants in the study were similar (*p* > 0.05) (Table 1).

### 3.2. Pelvic Floor Muscle Strength and Activation Values

The change in MOS values after training significantly increased in the second and third groups (*p* < 0.05), while the increase in MOS values in the group trained with verbal instructions was not statistically significant (*p* > 0.05). According to the results of muscle activation data assessed with vaginal BF-EMG, only in the second group did the muscle contraction value significantly increase after training (*p* < 0.05), while no significant difference was found in the other groups (*p* > 0.05) (Table 2).

### 3.3. The Number of Errors and Abdominal Muscle Activation Values

When looking at the number of errors after training, a decrease was observed in all three groups (*p* < 0.05), but there was no significant difference between the groups (*p* > 0.05). According to the BF-EMG data of abdominal muscle contraction during PFMC, a decrease in abdominal muscle contraction values was found in the second group after training (*p* < 0.05), while an increase was observed in the other groups, but no statistically significant difference was found (*p* > 0.05) (Table 3).

### 3.4. Between-Group Comparison of Errors

There was no significant difference between the groups in terms of the comparison of incorrect movements made during PFMC (*p* > 0.05) (Table 4).

### 3.5. Within-Group Comparison of Errors

The error in adductor muscle contraction decreased in the first and second groups (*p* < 0.05), while gluteal muscle contraction decreased in all three groups (*p* < 0.05). In the first group, no “yes” responses were observed for breath holding, so statistical tests could not be performed. In the second and third groups, no significant difference was found (*p* > 0.05). In the first and third groups, hyperventilation did not occur in the participants, so statistical tests could not be performed. In the second group, no significant difference was found (*p* > 0.05). In terms of straining, no within-group differences were found in any group (*p* > 0.05) (Table 5).

### 3.6. Statistical Power Analysis

Our study was conducted with 48 women (16 women per group). When examining the power analysis based on the error rates obtained after the training, a medium effect size (F = 0.458) was found between the groups. For this effect size, it was determined that our study achieved 81% power at a 95% confidence level. Additionally, when examining the power levels for the pre-training and post-training error rates in the groups, strong effect sizes were observed in all groups (Group 1: dz = 1.121; Group 2: dz = 0.895; Group 3: dz = 1.139).When the power of these changes was examined, it was found that the first group achieved 98.6% power, the second group achieved 91.7%, and the third group achieved 98.9% power.

## 4. Discussion

In our study, it was found that both perineometer and DVP (assessed with MOS) were equally effective in teaching PFMC, while all three techniques were effective in reducing incorrect movements. Additionally, in the EMG-BF measurements, it was found that only the DVP technique reduced abdominal contraction and significantly increased pelvic floor muscle activation values. As far as we know, this study is the first randomized controlled trial comparing methods for teaching PFMC and evaluating which technique is more effective in reducing errors made by women during these trainings.

Studies have reported that pelvic health and women’s health physiotherapists experience uncertainty about which method to use in clinical practice and research. Many PFMCT protocols have been used in the literature, but there is no consensus on a standard method. Furthermore, the errors (during PFMCT) have not been adequately assessed from both objective and subjective perspectives [4].

In one study, women were given only verbal instructions during the first week postpartum, and their PFMCs were assessed observationally. While 29% of the participants were unable to perform any PFMCs, 24% exhibited insufficient contractions. However, after the verbal instructions were given, the correct PFMC performance rate increased to 73.6% [26]. In our study, although comprehensive training was provided beforehand, no significant increase was observed in terms of MOS values and muscle activation data measured with BF-EMG in Group I. The difference between our study and the literature may be due to the fact that we used different and more objective evaluation methods. In a systematic review examining different physiotherapy methods for facilitating PFMC, vaginal palpation is recommended for correct PFMC due to the simultaneous feedback provided by the therapist and the stimulation through tactile stimulation [4]. Thus, it has been reported that an interactive approach can encourage individuals’ participation and learning [10]. In our study, the increase in the MOS values observed in PFMC in Group II may be a result of the training method applied with this stimulation technique, supporting the literature.

The evaluation of pelvic floor muscles using surface electrodes placed on the perineum may be influenced by the activity of other muscles, such as the gluteal muscles, obturator muscles, or anal sphincter, making it not an ideal assessment method. To assess pelvic floor muscle activation more accurately and objectively, an intravaginal probe is used due to its proximity to the vagina [27]. In our study, we assessed the participants’ pelvic floor muscle activation using EMG-BF with an intravaginal probe. According to the results we obtained, a significant increase in muscle activation values was observed in Group II (which received training only with DVP). This result supports the view that sensory stimulation provided through vaginal palpation may lead to more effective outcomes in training.

During internal focus, individuals tend to disrupt automatic control processes while trying to consciously control their movements. When the focus is on the effect of the movement, automatic reflex-type control can occur. This allows for the control of unconscious, involuntary movements while promoting more effective and efficient movements. Studies have indicated that external focus enhances learning, while internal focus does not negatively affect learning [13]. In our study, we obtained more effective results with the training provided using external focus, while with internal focus, we only observed a reduction in five incorrect movements. This suggests that, although training can have positive effects in both cases, the impact generated through external focus is greater.

In our study, we addressed a total of six incorrect movements. Five of these incorrect movements were assessed through inspection or palpation. To objectively determine the coactivation of abdominal muscles during PFMC, we preferred to use EMG-BF. For other muscles, as we did not have a multi-channel device to assess them simultaneously, we controlled them using palpation and inspection techniques. The core region is a three-dimensional cylindrical structure composed of the abdominal muscles in the front, paraspinal muscles in the back, oblique muscles on the sides, the diaphragm at the top, and the pelvic floor muscles at the bottom. These muscles respond to postural changes and external loads to maintain the body’s mechanical stability. EMG studies in the literature have shown that the pelvic floor muscles contribute to stabilization by contracting simultaneously with the transversus abdominis and diaphragm muscles [28]. While it is emphasized in the literature that abdominal muscles should relax during PFMC, performing the correct PFMC that activates the deep abdominal muscles without causing an increase in intra-abdominal pressure can be challenging. Additionally, it has been found that completely eliminating abdominal muscle activity reduces voluntary contraction of the pelvic floor muscles by 25%. Therefore, controlling and minimizing abdominal muscle activity during PFMC is important. Neumann and Gill found that it is not possible for women to fully contract their PFM without also contracting their transversus abdominis and internal oblique muscles [29]. They found that maximum PFM contraction could not be achieved without an increase in EMG activity in the lower part of the rectus abdominis [30]. Based on these findings, we observed a reduction in abdominal muscle co-contraction with training provided using only the DVP technique.

Pinheiro et al. emphasized that correct muscle contraction is as important as muscle strength itself and reported that 90% of the participants initially used accessory muscles. However, after the training, 90% of these women stopped using these muscles [6,10], and it was found that 216 out of 386 women (57%) contracted at least one other muscle group simultaneously during PFMC. Among these women, the most common muscle contractions were abdominal muscle contraction (*n* = 131; 35%), breath holding (*n* = 123; 33%), and gluteal muscle contraction (*n* = 84; 22%). In our study, before the training, we found the adductor error to be 62.5% in Group I, 62.5% in Group II, and 43.8% in Group III; gluteal error to be 68.8% in Group I, 56.3% in Group II, and 68.8% in Group III; and breath holding to be 25% in Group I, 25% in Group II, and 31.3% in Group III. Based on these data, among the five categories, gluteal muscle co-contraction, adductor muscle co-contraction, and breath holding were the most commonly observed errors. These results suggest that greater attention should be given to these areas during PFMT, as post-training, the values for these errors decreased significantly.

Our study had some limitations. One of the first limitations was that the physiotherapist administering the assessment and training could not be blinded to the treatment groups. To minimize the impact of this on the evaluation results, objective measurements were utilized in this study. Another limiting factor could be the use of a single-channel EMG-BF device, which only allowed for the evaluation of abdominal muscles. In future studies, we recommend evaluating other muscles with EMG-BF as well. Despite these limitations, the randomized controlled design and the use of both objective and subjective measurements are strengths of our study. This study, which highlights the importance of treatment planning in healthcare and the necessity of error-free pelvic floor muscle training, is the first to compare three different PFMCTs. Teaching and maintaining movement through feedback methods and providing proprioceptive input is crucial. Proprioceptive information can facilitate both the learning process of movement and the continuation of learned motor behaviors. The feedback methods we used in our study were also one of our strengths.

## 5. Conclusions

In our study, we observed that the training provided in three groups contributed to reducing common errors during pelvic floor muscle contraction training (PFMCT). However, the reduction in abdominal muscle co-contractions during pelvic floor muscle contraction was only significant in the DVP group. Additionally, we observed that the DVP group contributed to the formation of stronger pelvic floor muscle contractions. However, there were no differences between the groups. These results also emphasize the importance of evaluating incorrect movements during PFMCT. Future research will help develop more comprehensive strategies to improve pelvic floor health using these training methods. Planning follow-up studies lasting at least 3 months will be important in evaluating the long-term effects of this training and comparing their impact over time.

## Figures and Tables

**Table 1:** Comparison of participant baseline characteristics.

	Group I (*n* = 16)	Group II (*n* = 16)	Group III (*n* = 16)	
	Median (IQR)	Median (IQR)	Median (IQR)	*p* (Value)
Age (years)	46.5 (43.5–49.75)	43 (40–49)	44.5 (43–48.5)	0.382 (kw = 1.927) ^a^
BMI (kg/m^2^)	28.85 (24–30.25)	26.8 (24.1–29.35)	25.95 (22.53–29.48)	0.507 (kw = 1.36) ^a^
Gravida (*n*)	3 (2–3)	3 (2.25–4)	3 (2.25–4)	0.665 (kw = 0.817) ^a^
Parity (*n*)	2.5 (2–3)	3 (2–3)	3 (2–3)	0.986 (kw = 0.029) ^a^
Abortion (*n*)	0 (0–0)	0 (0–0.75)	0 (0–0)	0.745 (kw = 0.588) ^a^
Curretage (*n*)	0 (0–1)	0 (0–0.75)	1 (0–1)	0.23 (kw = 2.942) ^a^
Primary (*n*)	5 (%31.3)	5 (%31.3)	3 (%18.8)	
Education high school (*n*)	6 (%37.5)	5 (%31.3)	5 (%31.3)	0.826 (χ^2^ = 1.503) ^b^
Undergraduate and graduate (*n*)	5 (%31.3)	6 (%37.5)	8 (%50)	
Vaginal (*n*)	10 (%62.5)	13 (%81.3)	8 (%50)	
Mode of delivery Cesarian (*n*)	3 (%18.8)	2 (%12.5)	3 (%18.8)	0.356 (χ^2^ = 4.392) ^b^
Vaginal and Cesarian (*n*)	3 (%18.8)	1 (%6.3)	5 (%31.3)	

*p* < 0.05 indicates a statistically significant difference. Median (IQR): interquartile range ^a^: kw: Kruskal–Wallis variance analysis; ^b^: χ^2^ test: the chi-square test.

**Table 2:** Comparison of MOS values and muscle activation values with EMG-BF before and after PFMCT.

		Group I		Group II		Group III		Between-Group (*p*)
		A.M. ± S.D.	Med (IQR)	A.M. ± S.D.	Med (IQR)	A.M. ± S.D.	Med (IQR)	
MOS (pre-T)		2.56 ± 0.73	2 (2–3)	2.69 ± 0.7	3 (2–3)	2.5 ± 0.63	2 (2–3)	0.721 (kw = 0.654) ^a^
MOS (post-T)		2.69 ± 0.7	3 (2–3)	2.94 ± 0.57	3 (3–3)	2.88 ± 0.72	3 (2–3)	0.497 (kw = 1.399) ^a^
Within-group (*p*)		0.157 (z = −1.414) ^c^		0.046 * (z = 2) ^c^		0.014 * (z = −2.449) ^c^		
MOS difference		−0.13 ± 0.34	0 (0–0)	−0.25 ± 0.45	0 (−0.75–0)	−0.38 ± 0.5	0 (−1–0)	0.271 (kw = 2.611) ^a^
Vaginal EMG (pre-T) (cont)		34.03 ± 22.34	28.15 (17.88–37.88)	34.77 ± 23.3	26.85 (20.08–39.8)	30.03 ± 15.62	29.6 (19.13–35.93)	0.861 (kw = 0.298) ^a^
Vaginal EMG (post-T) (cont)		33.22 ± 28.33	25.4 (13.75–39.08)	39.46 ± 24.95	33.95 (24.2–46.13)	39.35 ± 38.45	29.2 (18.8–43.63)	0.285 (kw = 2.509) ^a^
Within-group (*p*)		0.379 (z = −0.879) ^c^		0.025 * (t = −2.498) ^d^		0.932 (z = −0.085) ^c^		
		*n*	%	*n*	%	*n*	%	*p*
Vaginal EMG difference	increase	5	31.3	11	68.8	7	43.8	0.097 (χ^2^ = 4.675) ^b^
	decrease	11	68.8	5	31.3	9	56.3	

* p < 0.05 indicates a statistically significant difference. A.M: arithmetic mean; S.D: standard deviation; median (IQR): interquartile range; ^a^: kw: Kruskal–Wallis variance analysis; ^b^: χ^2^: chi-square test; ^c^: z: Wilcoxon paired-sample test; ^d^: *t*-test for dependent groups; MOS: Modified Oxford Scale; (pre-T): pre-training; (post-T): post-training; (cont): contraction; EMG: electromyography.

**Table 3:** The number of errors made during PFMC and abdominal muscle activation data.

		Group I		Group II		Group III		
		A.M. ± S.D.	Med (IQR)	A.M. ± S.D.	Med (IQR)	A.M. ± S.D.	Med (IQR)	Between-Group (*p*)
tNoE (pre-T)		1.88 ± 1.67	2 (0–3.5)	1.69 ± 1.35	1.5 (1–2.75)	1.88 ± 1.02	2 (1–3)	0.802 (kw = 0.442) ^a^
tNoE (post-T)		0.25 ± 0.68	0 (0–0)	0.56 ± 1.15	0 (0–0.75)	0.81 ± 0.83	1 (0–1.75)	0.061 (kw = 5.595) ^a^
Within-group (*p*)		0.004 * (z = −2.848) ^c^		0.007 * (z = −2.684) ^c^		0.003 * (t = 3.597) ^c^		
Difference of tNoE		1.63 ± 1.54	2 (0–2.75)	1.13 ± 1.45	1 (0–2)	1.06 ± 1.18	1 (0–2)	0.57 (kw = 1.123) ^a^
Abd EMG (pre-T) (cont)		13.82 ± 10.39	8.65 (5.43–20.63)	18.75 ± 14.32	14.45 (8.38–20.48)	23.19 ± 17.18	18.85 (9.9–37.63)	0.177 (kw = 3.468) ^a^
Abd EMG (post-T) (cont)		17.15 ± 15.62	14.25 (5.35–23.25)	16.16 ± 13.84	10.7 (6.13–21.33)	26.48 ± 22.01	18.5 (8.95–40.73)	0.305 (kw = 2.378) ^a^
Within-group (*p*)		0.535 (z = −0.621) ^c^		0.016 * (t = 2.699) ^d^		0.532 (z = −0.625) ^c^		
EMG difference (cont)		3.33 ± 14.4	0.1 (−9.6–3.18)	2.59 ± 3.84	1.9 (−0.35–5)	3.29 ± 9.57	0.15 (−10.55–3.18)	0.139 (kw = 3.946) ^a^
		*n*	%	*n*	%	*n*	%	*p*
Abd EMG difference	increase	8	50	4	25	7	43.8	0.322 (χ^2^ = 2.265) ^b^
	decrease	8	50	12	75	9	56.3	

* p < 0.05 statistically significant difference, A.M: arithmetic mean, S.D: standard deviation, med (IQR): interquartile range, ^a^: kw: Kruskal–Wallis variance analysis, ^b^: χ^2^: chi-square test, ^c^: z: Wilcoxon signed-rank test, ^d^: t: dependent groups *t*-test tNoE: the number of errors, (pre-T): pre-training; (post-T): post-training; EMG: electromyography; Abd: abdominal muscles.

**Table 4:** Between-group comparison of errors made during PFMC.

		Group I	Group II	Group III	Between-Group (*p*)
Addcont pre-T	Yes	10 (%62.5)	10 (%62.5)	7 (%43.8)	0.467 (χ^2^ = 1.524)
	No	6 (%37.5)	6 (%37.5)	9 (%56.3)	
Addcont post-T	Yes	1 (%6.3)	2 (%12.5)	3 (%18.8)	0.552 (χ^2^ = 1.189)
	No	15 (%93.8)	14 (%87.5)	13 (%81.3)	
Glcont pre-T	Yes	11 (%68.8)	9 (%56.3)	11 (%68.8)	0.695 (χ^2^ = 0.729)
	No	5 (%31.3)	7 (%43.8)	5 (%31.3)	
Glcont post-T	Yes	2 (%12.5)	3 (%18.8)	3 (%18.8)	0.855 (χ^2^ = 0.312)
	No	14 (%87.5)	13 (%81.3)	13 (%81.3)	
SB pre-T	Yes	4 (%25)	4 (%25)	5 (%31.3)	0.901 (χ^2^ = 0.208)
	No	12 (%75)	12 (%75)	11 (%68.8)	
SB post-T	Yes	0 (%0)	2 (%12.5)	2 (%12.5)	0.181 (χ^2^ = 3.423)
	No	16 (%100)	14 (%87.5)	14 (%87.5)	
EI pre-T	Yes	1 (%6.3)	2 (%12.5)	3 (%18.8)	0.552 (χ^2^ = 1.189)
	No	15 (%93.8)	14 (%87.5)	13 (%81.3)	
EI post-T	Yes	0 (%0)	1 (%6.3)	0 (%0)	0.326 (χ^2^ = 2.24)
	No	16 (%100)	15 (%93.8)	16 (%100)	
St pre-T	Yes	4 (%25)	2 (%12.5)	2 (%12.5)	0.564 (χ^2^ = 1.146)
	No	12 (%75)	14 (%87.5)	14 (%87.5)	
St post-T	Yes	1 (%6.3)	1 (%6.3)	3 (%18.8)	0.433 (χ^2^ = 1.673)
	No	15 (%93.8)	15 (%93.8)	13 (%81.3)	

(pre-T): pre-training; (post-T): post-training; (cont): contraction; (SB): stop breathing; (EI) enhanced inhaling; (St): straining; Add: adductor muscles; Gl: gluteal muscles.

**Table 5:** Within-group comparison of errors made during PFMC.

Group			Addcont post-T		
			yes	no	Within-group (*p*)
Group I	addcont Pre-T	yes	1 (%6.3)	9 (%56.3)	0.004 *
		no	0 (%0)	6 (%37.5)	
Group II	addcont Pre-T	yes	2 (%12.5)	8 (%50)	0.008 *
		no	0 (%0)	6 (%37.5)	
Group III	addcont Pre-T	yes	3 (%18.8)	4 (%25)	0.125
		no	0 (%0)	9 (%56.3)	
Group			Glcont post-T		
			yes	no	Within-group (*p*)
Group I	Glcont Pre-T	yes	2 (%12.5)	9 (%56.3)	0.004 *
		no	0 (%0)	5 (%31.3)	
Group II	Glcont Pre-T	yes	3 (%18.8)	6 (%37.5)	0.031 *
		no	0 (%0)	7 (%43.8)	
Group III	Glcont Pre-T	yes	3 (%18.8)	8 (%50)	0.008 *
		no	0 (%0)	5 (%31.3)	
Group			SB post-T		
			yes	no	Within-group (*p*)
Group I	SB Pre-T	yes	(%0)	4 (%25)	-
		no	(%0)	12 (%75)	
Group II	SB Pre-T	yes	1 (%6.3)	3 (%18.8)	0.625
		no	1 (%6.3)	11 (%68.8)	
Group III	SB Pre-T	yes	2 (%12.5)	3 (%18.8)	0.25
		no	0 (%0)	11 (%68.8)	
Group			EI post-T		
			yes	no	Within-group (*p*)
Group I	EI Pre-T	yes	(%0)	1 (%6.3)	-
		no	(%0)	15 (%93.8)	
Group II	EI Pre-T	yes	1 (%6.3)	1 (%6.3)	1
		no	0 (%0)	14 (%87.5)	
Group III	EI Pre-T	yes	(%0)	3 (%18.8)	-
		no	(%0)	13 (%81.3)	
Group			St post-T		
			yes	no	Within-group (*p*)
Group I	St Pre-T	yes	1 (%6.3)	3 (%18.8)	0.25
		no	0 (%0)	12 (%75)	
Group II	St Pre-T	yes	0 (%0)	2 (%12.5)	1
		no	1 (%6.3)	13 (%81.3)	
Group III	St Pre-T	yes	1 (%6.3)	1 (%6.3)	1
		no	2 (%12.,5)	12 (%75)	

* *p* < 0.05 statistically significant difference; McNemar test, (pre-T): pre-training; (post-T): post-training; (cont): contraction; (SB): stop breathing; (EI) enhanced inhaling; (St): straining; Add: adductor muscles; Gl: gluteal muscles.

## Data Availability

The datasets used and/or analyzed during the current study are available from the corresponding author upon reasonable request, due to ethical committee restrictions.
